# Mapping autism’s research landscape: trends in autism screening and its alignment with sustainable development goals

**DOI:** 10.3389/fpsyt.2023.1294254

**Published:** 2024-02-01

**Authors:** Prema Nedungadi, Selina Marianna Shah, Mark Andrew Stokes, Vinith Kumar Nair, Ajit Moorkoth, Raghu Raman

**Affiliations:** ^1^Amrita School of Computing, Amrita Vishwa Vidyapeetham, Kollam, India; ^2^Amrita Vishwa Vidyapeetham, Amritapuri, India; ^3^Faculty of Health, Deakin Univeristy, Burwood, VIC, Australia; ^4^Seed Special Education Center, Dubai, United Arab Emirates; ^5^Amrita School of Business Amritapuri, Amrita Vishwa Vidyapeetham University, Coimbatore, Tamil Nadu, India

**Keywords:** autism spectrum disorder, ASD, SDG, neurodevelopment, bibliometrics, disability, mental health, ASD identification

## Abstract

**Introduction:**

Autism Spectrum Disorder is a complex neurodevelopmental syndrome that profoundly affects social interactions, communication, and sensory perception. The research traced the evolution of autism research from 2011-2022, specifically focusing on the screening and diagnosis of children and students.

**Methods:**

Through an analysis of 12,262 publications using the PRISMA framework, bibliographic coupling, science mapping, and citation analysis, this study illuminates the growth trajectory of ASD research and significant disparities in diagnosis and services.

**Results:**

The study indicates an increasing trend in autism research, with a strong representation of female authorship. Open Access journals show a higher average citation impact compared to their closed counterparts. A keyword co-occurrence analysis revealed four central research themes: Child Development and Support Systems, Early Identification and Intervention, Prevalence and Etiology, and Mental Health. The pandemic’s onset has prioritized research areas like mental health, telehealth, and service accessibility.

**Discussion:**

Recommendations on a global level stress the importance of developing timely biological markers for ASD, amplifying Disability Inclusion research, and personalizing mental health services to bridge these critical service gaps. These strategies, underpinned by interdisciplinary collaboration and telehealth innovation, particularly in low-resource settings, can offer a roadmap for inclusive, context-sensitive interventions at local levels that directly support SDG3’s aim for health and well-being for all.

## Introduction

Autism spectrum disorder (ASD) is a neurological condition that affects how a person perceives their environment (1). Each case is unique (2). Difficulty in social interaction and repetitive behavior patterns are two distinct characteristics of ASD (3). In the past 12 years, ASD research has come to the fore from a global perspective due to the increase in the perceived prevalence of Autism (4), parent advocacy (5), and potentially the significance of equity and inclusion in the United Nations Sustainable Development Goals (SDG) (6). The pandemic raised the need for more ASD research (7–9).

Studying the relationship between ASD and the SDGs identifies the challenges faced by people with ASD and ways to address them. The SDG, including SDG 3 (Good Health and Well-Being), SDG 4 (Quality Education), SDG 8 (Decent Work and Economic Growth), and SDG 10 (Reduced Inequalities), need to be specifically addressed in the context of ASD. These SDGs are particularly relevant as they address the health, education, employment, and social equality issues faced by individuals on the autism spectrum. Research on this topic can help to identify gaps in the current interventions and services provided to individuals on the autism spectrum and inform the development of more effective strategies to support them.

There are a range of diagnostic and screening tools used across the globe. The criteria are predominantly based on the DSM-5-TR (The Diagnostic and Statistical Manual of Mental Disorders, 5th edition, test revision) and the ICD-11 (International Classification of Diseases, 11th edition). Both are dynamic and changing, the former is used in clinical diagnosis and research, and the latter also serves insurance and legal purposes (10). Both DSM-5 (11) and ICD-11 (12) classify ASD under neurodevelopmental disorders (13) and are characterized by impairments in the growth and development of the human brain (14). Global statistics for children diagnosed with ASD vary significantly across countries, diagnostic tools, cultures and socioeconomic groups, ranging from 0.3/100 in Southeast Asia, to 2/100 in the Western Pacific (15) and 1/36 in the USA (16). The WHO (World Health Organization) quotes evidence from Zeidan (15) of 1 in 100 as an estimated average figure for the global prevalence of autism, with the proviso that the prevalence of autism could be higher. Prevalence is defined as the proportion of the population that is autistic (17). Data from national databases and studies examining the prevalence of autism in the United States (18), the United Kingdom (19), Denmark (20), and Sweden (21) indicate a significant increase in autism prevalence rates over the past decade. Several contributing factors to this rise include the changes to and broadening of diagnostic criteria, a shift of mental health diagnoses to autism, challenges related to healthcare accessibility, increased awareness and expertise in the community and field of autism, environmental influences, and cultural aspects.

A study by Salari et al. (22) proposes a different perspective, suggesting that in the USA there may have been a “plateau” in autism incidence (the total number of new cases each year) since 2016, which could potentially be observed in other countries as well. Russell et al. (19) study in the UK shows the incidence of autism of 787% between 1998 and 2018; in 1998 1 adult in 100,000 was diagnosed with autism, with this figure rising to 20 in 100,000 in 2018. One causing factor could be that autistic individuals who are cognitively able tend to be diagnosed later as they tend to present later (19). As early diagnosis becomes more accessible, it could follow that the rise in incidence among adult’s levels off and reaches a plateau.

Gender diagnosis ratios have changed, with recent figures suggesting reports that 3:1 is more accurate (23). Lockwood Estrin et al. (24) suggest that gender bias in diagnosis accounts for differences between females receiving later diagnoses and males. Research continues to highlight the potential that early screening and diagnosis, resulting in early interventions, can positively impact the functionality and reduce the consequent support needed for many autistic individuals (25, 26).

This study employs bibliometric analysis to examine research on ASD, specifically looking at diagnosing and screening aspects of children. Through two basic methods—performance analysis and science mapping—bibliometric analysis can be used to assess a particular scientific subject using bibliographic data (27, 28). This method is frequently employed to determine the development of a specific scientific field (29). Previous systematic and bibliometric studies on ASD have covered various aspects such as characterizing patients (30), evaluation and treatment of children and adolescents (31), challenging behaviors in children with ASD (32), evidence-based assessment of ASDs (33), and a systematic review of technologies used for screening and assessing ASD in children (34). Sweileh et al. (35) analyzed 18,490 publications from 2005 to 2014 and found a progressive annual growth in ASD research, with US academic and research institutions dominating the top 10 list of productive institutions. Carmona-Serrano et al. (36) analyzed 5,512 publications on autism in education from 1963 to 2019. Shekarro et al. (37) conducted a bibliometric analysis of executive functions in ASD, studying 5,514 publications between 1990 and 2019 and finding that the United States has the highest number of publications. Luor et al. (38) analyzed 95 studies and looked at 55 journals using the Social Sciences Citation Index (SSCI) from 1998 to 2020. Even though it is not listed among the DSM-5 ASD criteria, they found that the term Asperger syndrome (AS) is still used in academic research.

Though there have been bibliometric studies on the topic of ASD research, the context of SDG and the impact of COVID-19 on ASD research is missing, revealing a gap in research. This comprehensive bibliometrics study of ASD addresses these gaps, focusing on screening and diagnostic aspects of children with ASD.

This study explores the following research questions (RQ).

1. What are the trends in publications and citations in ASD research?
2. What is the trend of Open Access (OA) publications?
3. Is there a gender disparity in authorship?
4. Which are the most productive institutions?
5. Which are the top contributing countries?
6. What is the intellectual structure regarding countries, journals, and keywords?
7. How well does ASD research align with the SDG?
8. What has been the impact of COVID-19 on ASD research?

The remaining sections are arranged as follows: The study methodology, including bibliometric analysis and the PRISMA (Preferred Reporting Items for Systematic Reviews) framework, is covered in the first section. The second part is about the results and discussions of the study and has two subparts. Trends of publications, citations, open access, most productive institutions, top contributing countries, gender representation, best-cited journals, and keyword co-occurrence networks are initially discussed. After that, the influence of COVID-19 and how ASD research aligns with the SDGs are discussed. Finally, conclusions are drawn with future research directions and limitations.

## Methods

A systematic literature review investigates ASD publications to answer the objectives and research questions. Various bibliometric techniques are used as part of the study.

### Bibliometric analysis

The use of bibliometric analysis in this study aimed to identify emerging trends and delve into the intellectual structure of the field of ASD (ASD) in the extant literature (39). This was achieved by applying science mapping methods like bibliographic coupling and keyword co-occurrence analysis.

The term “bibliographic coupling” (40) refers to two documents referring to the same third document, and it is frequently used to measure document similarity (27, 41, 42). These techniques provide valuable insights into the relationships between different research works and spot trends and prevalent themes in the field (43, 44).

The Scopus database was used to conduct the literature search. Scopus is also associated with high-quality meta-data (45). This study employs a bibliometric mapping approach and uses the VOS Viewer software to visualize the diverse networks generated from co-citation analyses (46, 47).

### PRISMA framework

**Figure 1:**
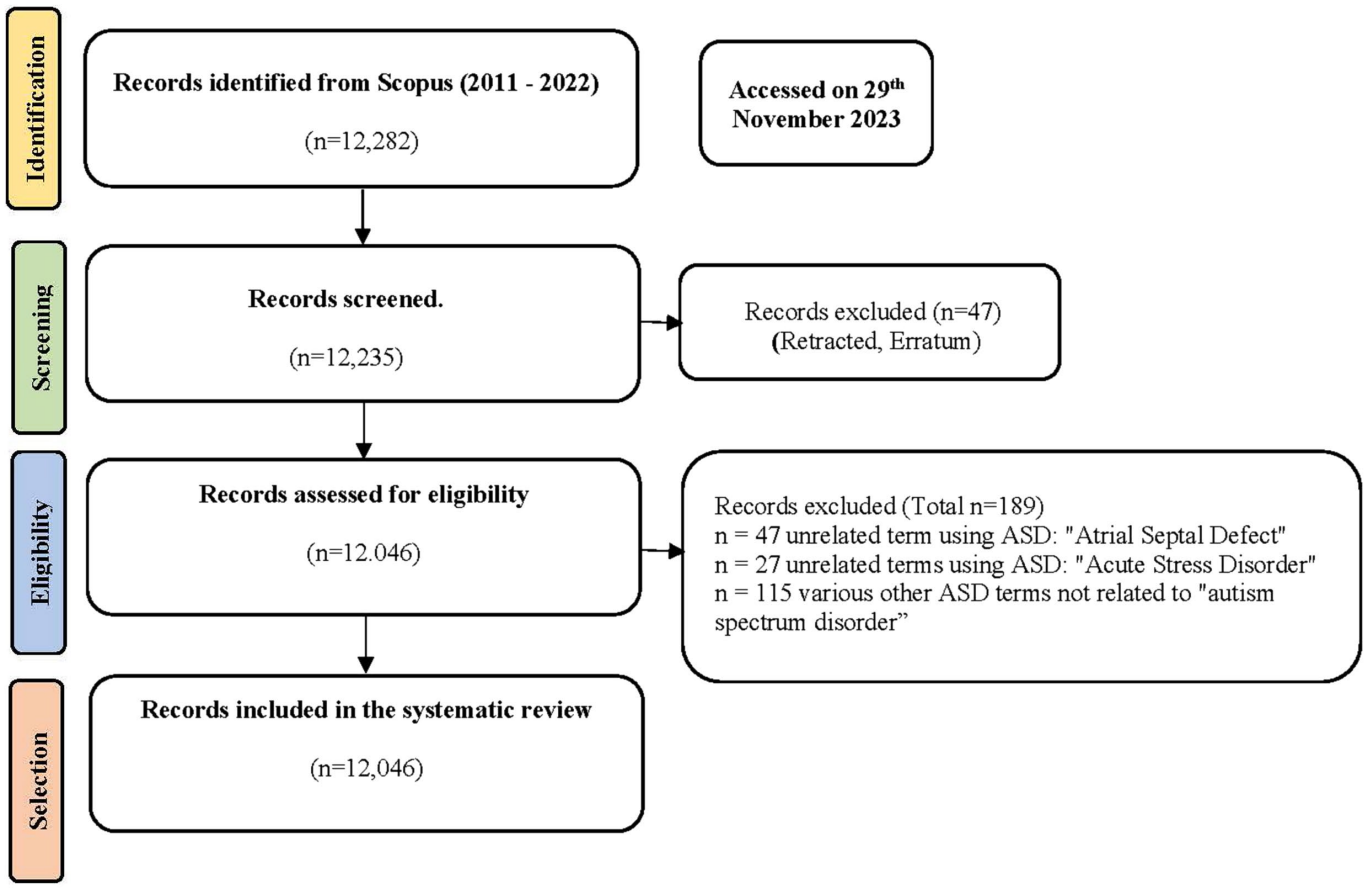
PRISMA framework.

The PRISMA framework is a recognized standard for conducting evidence-based systematic reviews and meta-analyses. The PRISMA statement consists of four essential phases: identification, screening, eligibility, and inclusion (see Figure 1).

The first step in the process was to choose the appropriate databases, and we selected Scopus as the source. Scopus is a unique database that requires articles to have a specific digital object identifier (DOI). The PRISMA framework (48), which has four stages: identification, screening, eligibility, and inclusion, was used in the next phase. This framework has been applied in several previous studies on ASD and has proven effective (36, 49, 50). The search term used in Scopus was TITLE-ABS (autism OR and OR autistic) AND TITLE-ABS (screen* OR diagn*) AND TITLE-ABS (student OR child*) AND (PUBYEAR >2010) AND (PUBYEAR <2023) AND (EXCLUDE (DOCTYPE,"tb”) OR EXCLUDE (DOCTYPE,"er”)).

The flow diagram (Figure 1) illustrates the study’s methodology based on the PRISMA-P framework.

## Results and discussion

### Trends of publications and citations

RQ1 is addressed in Table 1, which shows the progression of total publications (TP) and total citations (TC) in the field of ASD research from 2011 to 2022. The data indicates a steady growth in both TP and TC over the years, with a sharp increase in 2021 and 2022. The overall TP and TC growth rate indicate the trend within the research field. For example, the average growth rate of TP from 2011 to 2022 is approximately 10.9%. This shows that the research on ASD screening related to children is growing, and the impact of the research is also increasing.

**Table 1:** Trends of publications and cumulative citations.

	Open access			Closed access		
Year	TP	TC	TC/TP	TP	TC	TC/TP
2011	160	14,209	88.81	339	14,801	43.66
2012	195	15,899	81.53	428	17,015	39.75
2013	270	22,944	84.98	450	14,482	32.18
2014	293	20,657	70.5	484	14,821	30.62
2015	368	17,633	47.92	474	13,226	27.9
2016	409	15,710	38.41	499	9,964	19.97
2017	444	17,950	40.43	499	8,560	17.15
2018	474	15,421	32.53	508	7,325	14.42
2019	603	15,320	25.41	583	6,745	11.57
2020	718	13,287	18.51	628	4,805	7.65
2021	907	8,797	9.7	650	2,972	4.57
2022	946	4,830	5.11	716	1,444	2.02

TP, total publications; TC, total citations.

### Trends in open access

**Figure 2:**
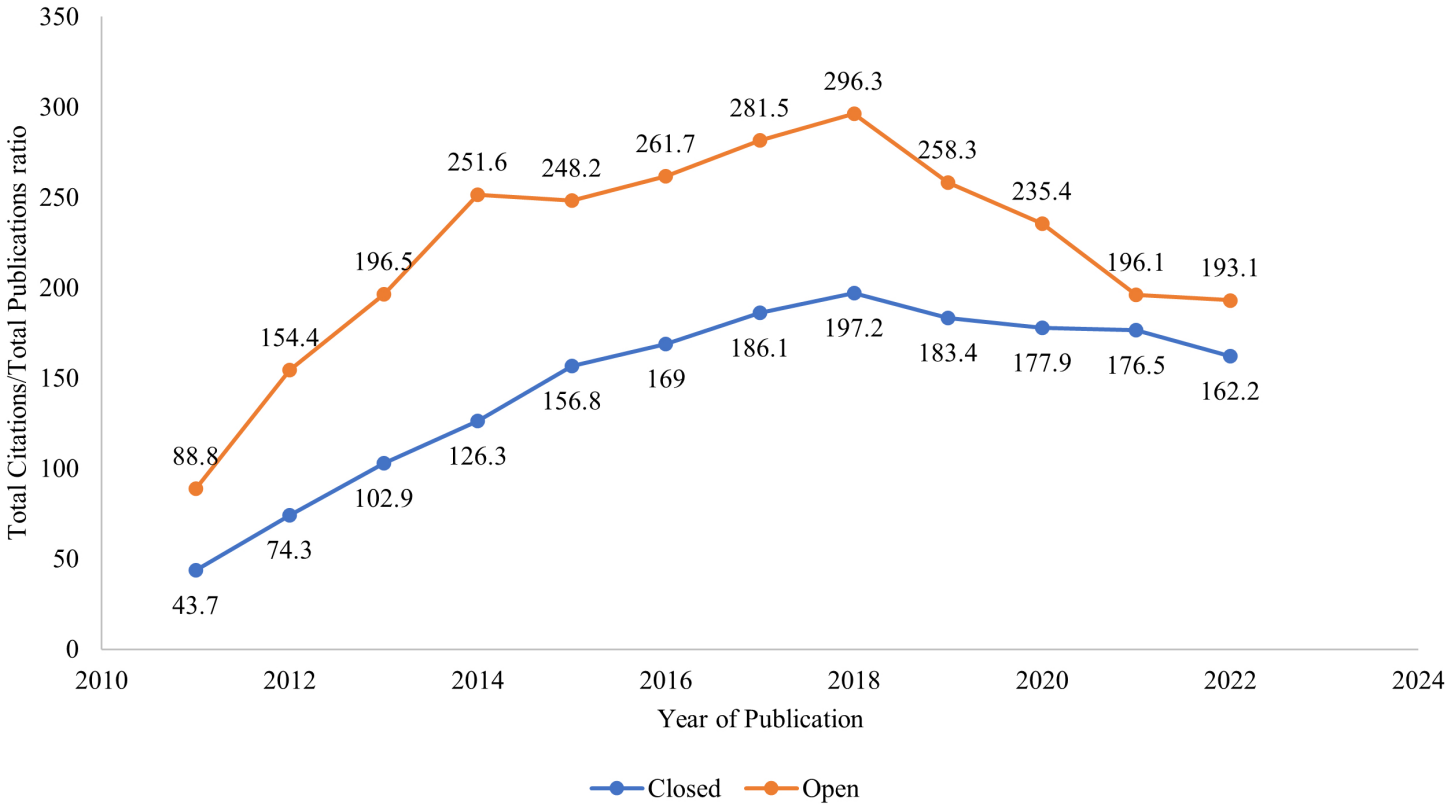
Open access publication trends.

Figure 2 shows the average citation effect of Open Access (OA) and closed publications from 2011 to 2022, addressing RQ2. Closed publications are higher than OA publications (6,716 vs. 5,545). A higher average citation per publication (TC/TP) of 27.2 can be observed for OA publications. For closed-access publications, this ratio is 16.2.

Figure 2 also shows the percentage trend of the publications of OA journals from 2011 to 2022. The curve shows an increasing trend from 2016 to 2021, concurring with similar results (51). The percentage of open-access publications has gone up from 42.3 to 55% in 2021, with a slight drop in 2022.

### Gender representation in authorship

Despite strides made toward promoting gender equality, disparities still exist in the global academic profession (52). These disparities extend to various scientific areas, including publication patterns (53). To investigate gender-based disparities in authorship and to address RQ3, the gender distribution of authors contributing to ASD was examined. Using the Gender API (54), the initial author’s gender was identified (55).

Though the gender identification tools have limitations, a study by Sebo (56) observed NamSor and Gender API as the most accurate tools. This research, utilizing NamSor, Gender API, Wiki-Gendersort, and genderize.io, underscored three primary sources of gender misclassification. Most errors were associated with unisex first names. Another category of mistakes was linked to non-western first names, and many errors were related to unique or uncommon first names. The author’s name, researcher id, affiliation, and publication year were obtained from the Scopus database.

Twelve thousand one hundred and ninety first author names were returned and further analyzed.

**Figure 3:**
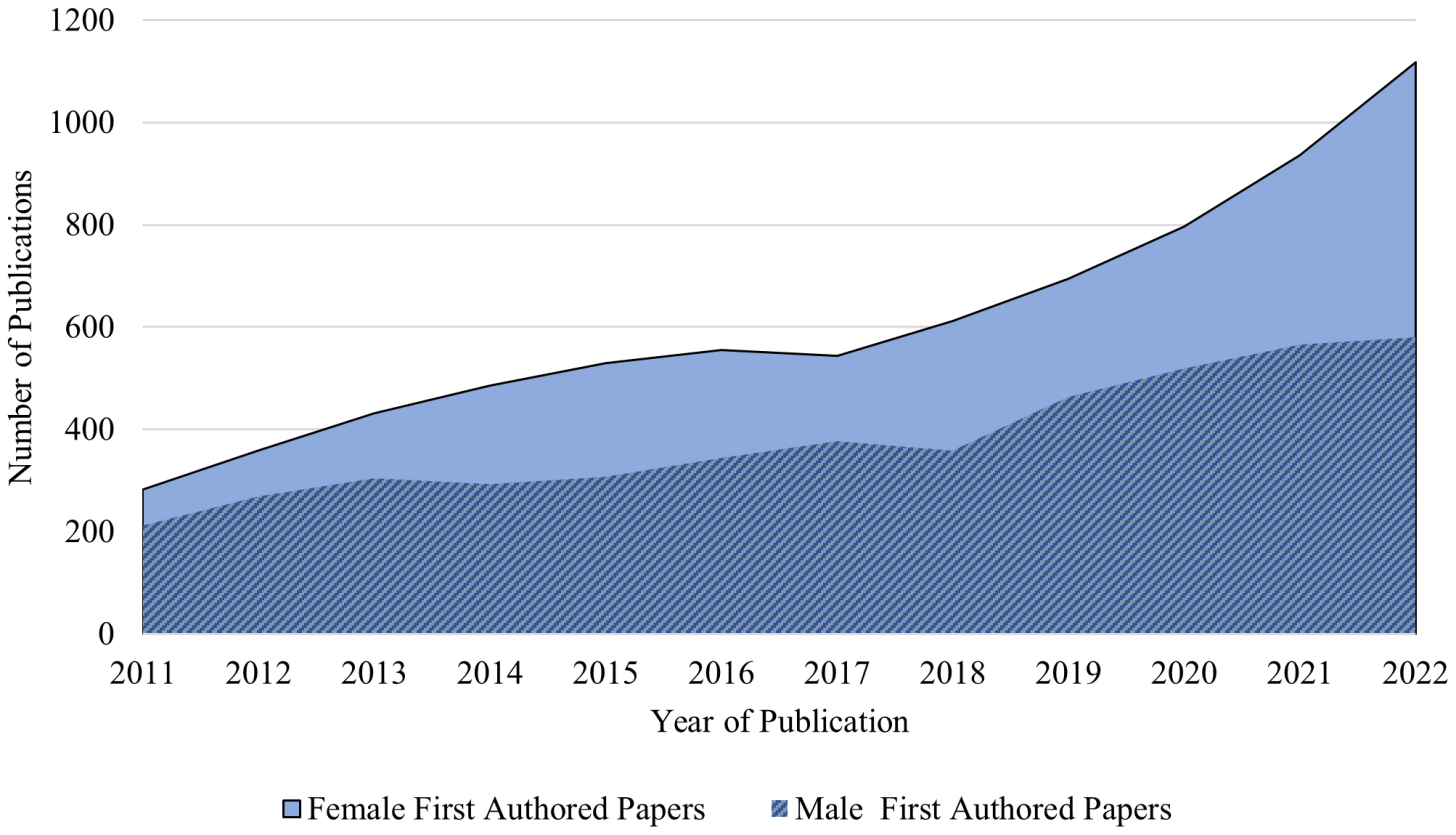
Year-wise proportion of male and female first-authored papers in ASD research.

Each record’s gender is determined using the Gender-API (54) based on the first name and the country. The final number of authors was 8,999 because the gender accuracy threshold was 95%. The annual distribution of first-authored papers in ASD research by men and women is shown in Figure 3. Between 2011 and 2022, the proportion of ASD papers with female first authors increased from roughly 56.6 to 65% (Table 2).

**Table 2:** Proportion of male and female authors by country.

Countries with the most number of authors (top 500 authors)	% of authors	% of male authors	% of female authors
United States	54.4%	34.93%	65.07%
United Kingdom	13.2%	48.48%	51.52%
Canada	6.4%	25%	75%
Australia	3.8%	15.79%	84.21%
Sweden	3.4%	41.18%	58.82%
	male authors	Female authors	Unknown
% of males and females in the top 500 authors (threshold 95%, total authors-406)	38%	62%	0%
% of publications	37.7%	60.3%	0.72%
% of citations	57.4%	41%	0.45%

### Best cited journals

Table 3 indicates the best-cited journals for ASD research according to TC, which indicates influence since the journal articles are recognized by authors (57). The best three journals based on TC are the Journal of Autism and Developmental Disorders, Pediatrics, and Autism, with 22,386, 11,319, and 9,952, respectively. The Journal of Autism and Developmental Disorders has the highest TP of 756. Nature has the largest TC/TP (986.6), followed by MMWR Surveillance (690). Autism is interdisciplinary, the other three being psychology/neuroscience/medical. All journals have a Scimago Journal Ranking (SJR) of Q1 or over 75% percentile.

**Table 3:** Best-cited journals.

Name	TC	TP	TC/TP	Scopus percentile
Journal of Autism and Developmental Disorders	25,402	707	35.93	85th
Pediatrics	12,913	110	117.39	98th
Autism	11,717	353	33.19	94th
Autism Research	9,309	355	26.22	79th
Journal of Child Psychology and Psychiatry and Allied Disciplines	8,886	134	66.31	98th
Research in Autism Spectrum Disorders	8,545	364	23.48	73rd
PLoS ONE	5,325	115	46.3	87th
Research in Developmental Disabilities	3,723	147	25.33	73rd
Journal of Developmental and Behavioral Pediatrics	2,971	138	21.53	61st
Frontiers in Psychiatry	1,369	143	9.57	71st

TP, total publications; TC, total citations; TC/TP, total citations per publication.

**Figure 4:**
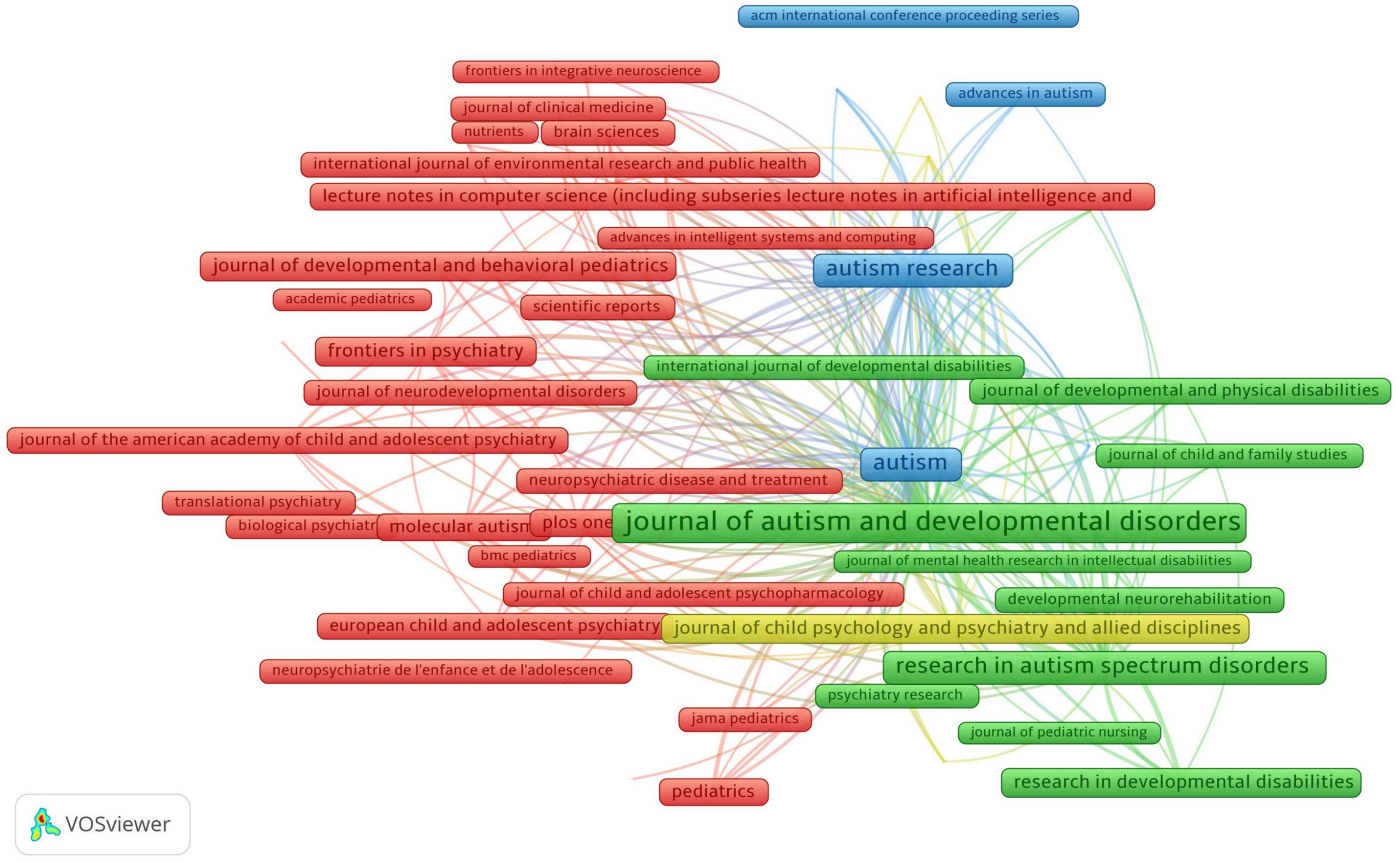
Journal clusters pathophysiology.

Further, this research analyzed the bibliographic coupling of journals (RQ6) that allows for identifying clusters and networks among documents sharing common references (58). Figure 4 shows the bibliographic coupling of journals. The minimum requirement for journal inclusion was set at 20 publications that met our search criteria. Out of 3,132 journals, 80 fulfilled this condition. The total number of publications, the number of citations they obtained, and the strength of the bibliographic couplings with those of other articles were calculated. Each bubble signifies a journal. Each bubble in the figure signifies a journal, where the bubble size represents the volume of publications, the link width indicates the count of shared citations between journals, and the color corresponds to the cluster.

The Journal of Autism and Developmental Disorders, shown in green (cluster 2), has the biggest bubble size, maximum link strength, total publications, and total citations among all the 80 journals which met the threshold.

Cluster 1 (red) has 53 journals, with the frontiers in psychiatry having the maximum link strength, followed by research in molecular autism. Cluster 1 comprises prominent journals in pediatrics, child adolescent psychiatry, and autism screening. These journals have strong connections, and high citations, indicating their influence in the field.

Cluster 2 (green) has 28 journals, with “The Journal of Autism and Developmental Disorders” journal having the maximum link strength and followed by Autism Research. The focus on autism research and developmental disorders in this cluster suggests the research interest in the challenges faced by ASD individuals. It highlights the ongoing efforts to advance knowledge and support in this area. Journals in this cluster contribute to understanding autism and related fields, although with slightly fewer links and citations compared to Cluster 1.

**Figure 5:**
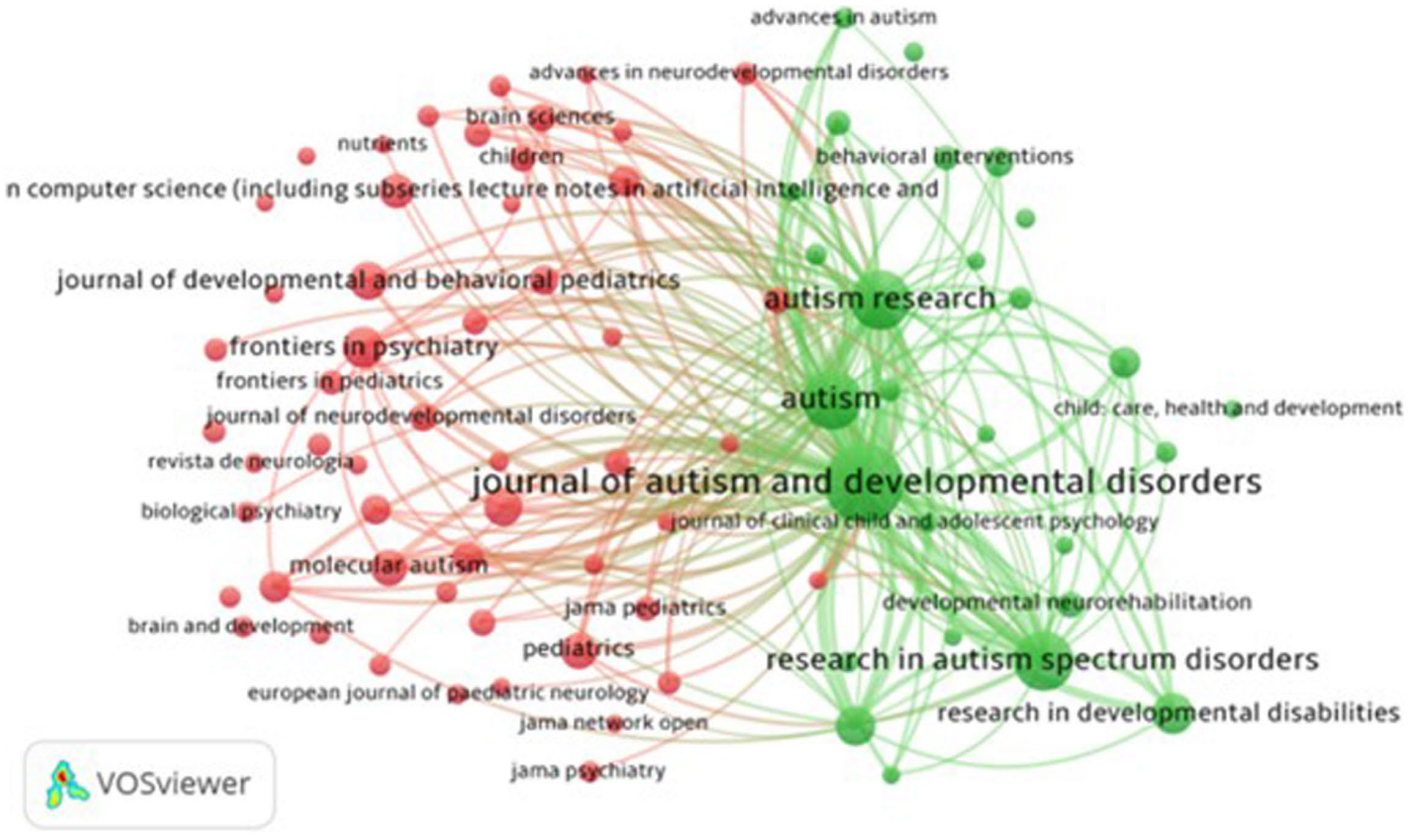
Temporal dynamics of journal sources.

The publication characteristics in the two clusters reflect different temporal dynamics (Figure 5) within the respective fields. Cluster 1 has more recent publications suggesting active interest, evolving research, and recent advances in autism, while Cluster 2 spans a range of publication years indicating earlier studies to contemporary investigations.

### Most productive institutions

Table 4 illustrates the most productive institutions that address RQ4. Six of these organizations are from the United States, while Sweden has two, and the United Kingdom and Canada have one each. Harvard University, King’s College London, and Karolinska Institute have the highest TP and TC.

**Table 4:** Most productive institutions.

Name	Country	TP	TC	TC/TP
Harvard Medical School	United States	268	13,753	51.32
King’s College London	United Kingdom	253	16,772	66.29
Karolinska Institute	Sweden	217	10,863	50.06
The University of California, Davis	United States	215	12,282	57.13
University of Toronto	Canada	207	9,974	48.18
University of North Carolina, Chapel Hill	United States	199	14,518	72.95
University of California, Los Angeles	United States	168	9,198	54.75
Goteborgs Universitet	Sweden	165	5,604	33.96
Boston Children’s Hospital	United States	164	7,808	47.61
The Children’s Hospital of Philadelphia	United States	160	8,273	51.71

TP, total publications; TC, total citations, TC/TP, total citations per publication. The top three universities based on TC/TP are the University of North Carolina, Chapel Hill (63.6), King’s College London (55.7), and the University of California, Davis (50.6).

### Most productive countries

The top contributing countries are addressed in Table 5 (RQ5), alongside prevalence data from two major studies. While United States is the leading contributing country in terms of quantity, with the largest percentage of TP and TC, the country with the highest TC/TP is Sweden, followed by Canada and the United Kingdom. Sweden, USA, and Canada also appear to consistently have the highest prevalence rates.

**Table 5:** Top contributing countries and prevalence rates.

Country	TP	TC	TC/TP	% of TP	Prevalence rates (per 10,000)	
					Zeidan et al. (15) and Özerk (60)*	Talantseva et al. (59)
United States	5,008	178,893	35.72	41.57	180–191	112 (92–133)
United Kingdom	1,242	50,516	40.67	10.31	30–190	67 (45–92)
Australia	682	22,210	32.57	5.66	150–250	41–45
Canada	670	27,650	41.27	5.56	104–136	71 (46–101)
China	621	10,101	16.27	5.16	10–39	39 (18–68)
Italy	530	13,883	26.19	4.4	52–134	95.3
Sweden	402	16,477	40.99	3.34	206–214	90 (59–127)
India	390	3,123	8.01	3.24	140	23
France	354	6,308	17.82	2.94	57–227*	–
Spain	312	6,565	21.04	2.59	40–87	32 (12–61)

*Source: Özerk (60). TP, total publications; TC, total citations; TC/TP, total citations per publication; %TP, percentage of total publications.

Prevalence, an epidemiological metric, is the proportion of a population affected by autism at a given time. The variability in prevalence rates across countries may be partly attributed to different research methodologies and different years of research. Differing autism prevalence rates across countries suggest examining the interplay of awareness, healthcare access, and research.

The wide range of prevalence in some countries like Australia, UK, and France may suggest diverse sub-populations that have different rates of prevalence or differences in research focus, healthcare practices across the country or access to care. High prevalence rates in Sweden and the United States might reflect a combination of higher incidence, greater awareness, better screening, and/or better reporting mechanisms.

Lower TC/TP ratios and lower prevalence rates may suggest less research, awareness and practice and/or differences in healthcare systems and screening or reporting practices.

**Figure 6:**
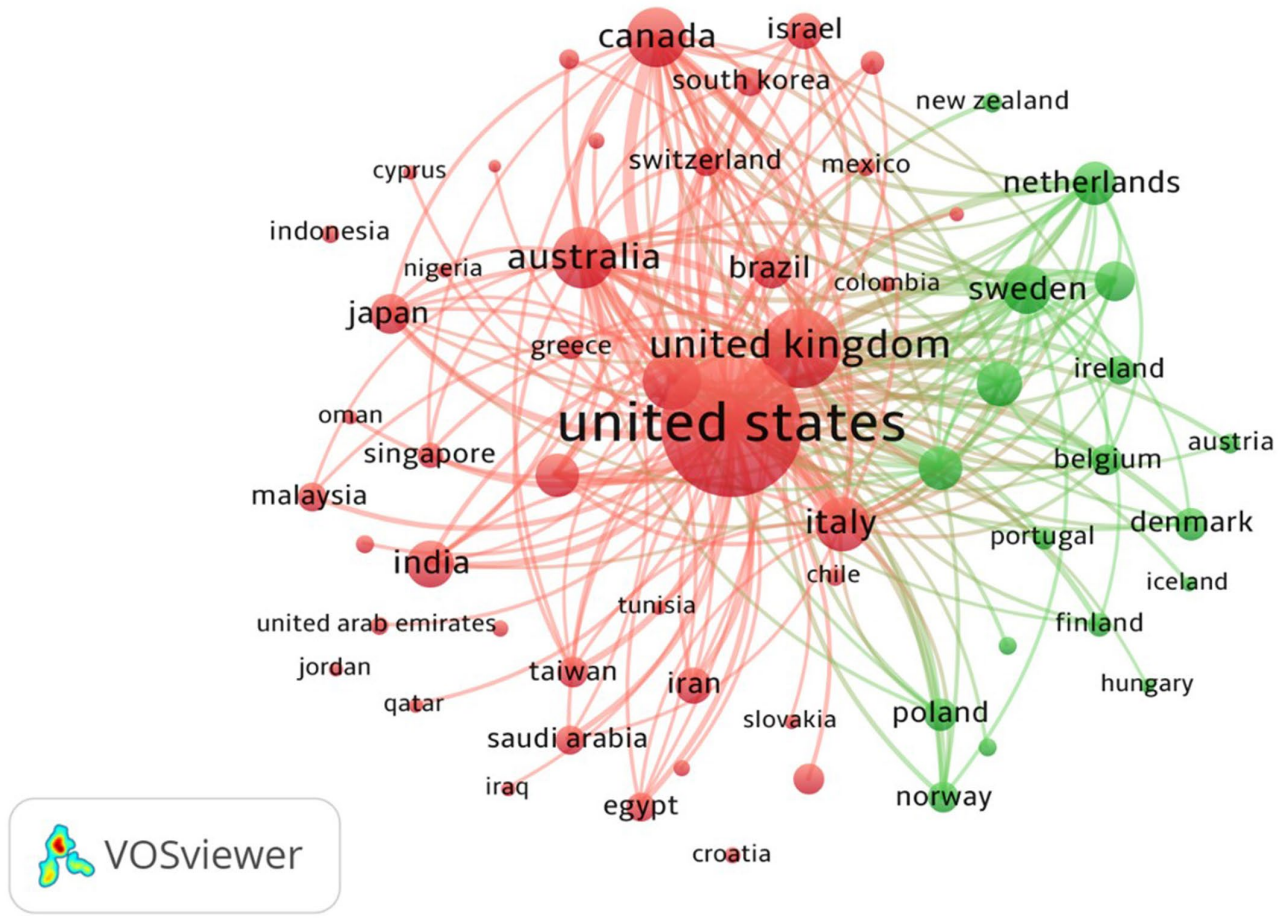
Bibliographic coupling of countries.

Further, we analyzed the bibliographic coupling of countries (RQ6) that occurs when publications from two countries reference publications from a third country. Bibliographic coupling is a systematic quantitative methodology that, by its transparent nature, adds value compared to qualitative approaches (61). Shared citations among studies can provide a valuable indicator of interlinkages with the same topic (62). Figure 6 offers a network visualization of the bibliographic coupling of countries.

Only countries with at least 20 publications were considered. Out of 301 countries, 88 satisfied this criterion. Each bubble in the figure stands for a country. The size of each bubble is representative of the total count of publications, while the width of the link indicates the volume of publications from two countries that cite works from other countries. The color corresponds to a cluster.

We see two clusters in Figure 6. Publications from countries within the same cluster cite one another with higher frequency. Cluster 1 (red), the largest cluster, has 44 countries, followed by Cluster 2 (green), with 18 countries. This suggests that the United States significantly influences ASD and that other countries are coupled with it. However, the figure also illustrates frequent coupling among countries such as Australia, Japan, the United Kingdom, and Italy.

In cluster 1 (red) United States has the most publications (TP:5,099), and strong links can be seen to developing countries like India, Japan, and Australia. In cluster 2 (green), Sweden has the highest TP (409) in this group, and strong links can be seen to the Netherlands, Ireland, Belgium, and Austria.

### Thematic clusters from keyword co-occurrence network

**Figure 7:**
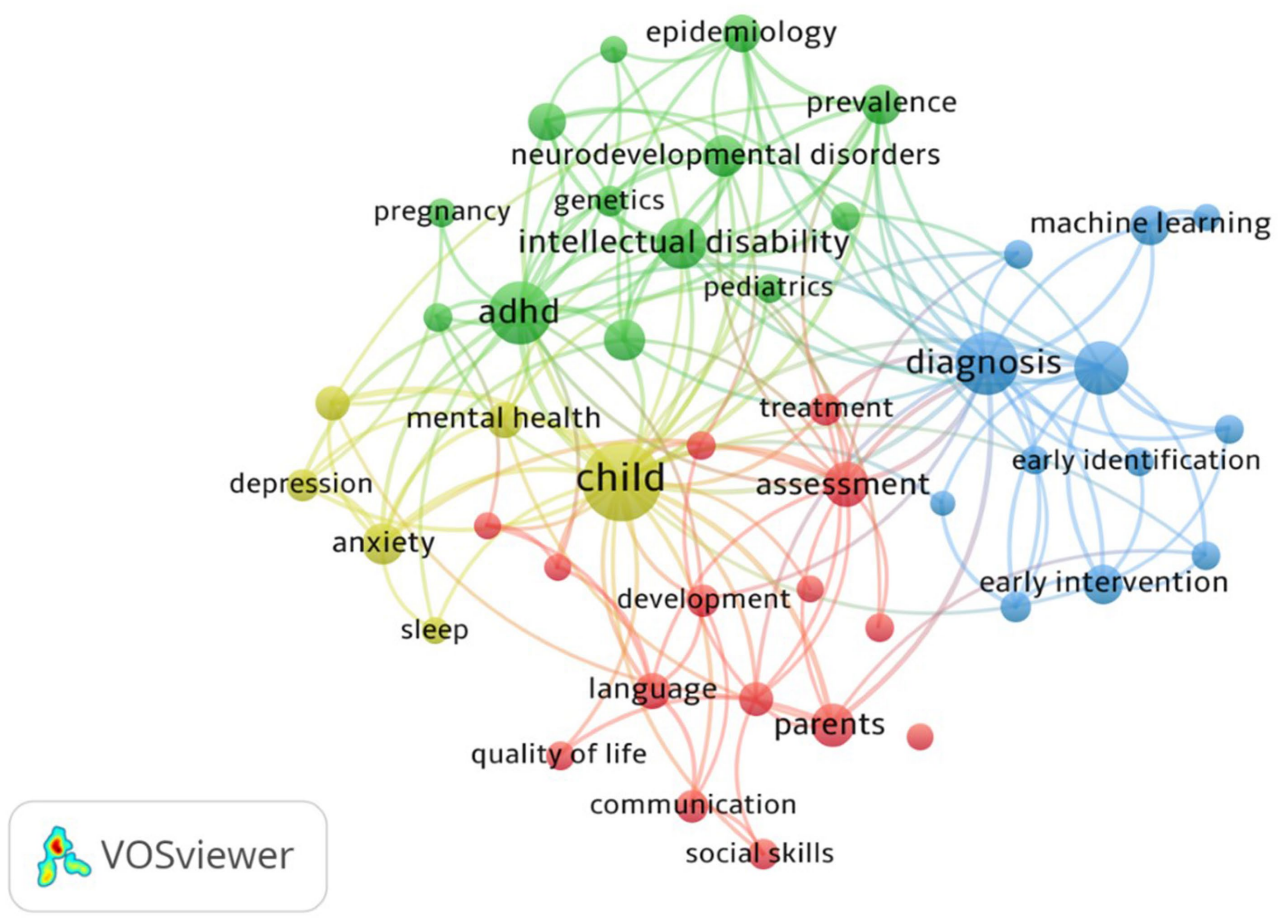
Keyword co-occurrence network.

To identify themes in ASD research, we conducted a co-occurrence network analysis of keywords (45) (RQ6). The network represents a domain’s collective knowledge. Based on patterns and the intensity of links between keywords that exist in the literature; it aids in the discovery of significant knowledge components and insights. The keyword organization shows the thematic cluster’s scope and essence (39). The network of keyword co-occurrences is shown in Figure 7.

Figure 7 presents the keyword co-occurrence network. Every keyword is shown as a bubble, and the size represents the frequency count of the keywords. The keywords’ weight determines the size of the label and the bubble. The color of a keyword corresponds to the cluster in which the keyword is found. The lines in the network denote the distance between two keywords and indicate the relatedness of the keywords in terms of links. The closer the two keywords are located, the stronger the relatedness between keywords.

Cluster 1(Red) ASD Child Development and Support Systems with keywords: *Asperger Syndrome, assessment, behavior, cognition, communication, development, education, intervention, language, parents, quality of life, social communication, social skills, systematic review* and *treatment*.

The main areas of concern are social communication, relationships, and repetitive behaviors (63). Individuals with autism may experience a range of cognitive and language disabilities, including language delays, cognitive defects, and attention and compulsion problems (64). However, Asperger’s Syndrome, absorbed into the DSM-5 definition of ASD in 2013, indicates an absence of language delay and cognitive issues and typically involves fewer support needs (65). Special education, school support, and early intervention are vital in helping achieve their full potential.

Interventions primarily fall into Comprehensive Treatment Models or focused interventions (66). Parents’ approaches influence the social adjustment, cognitive growth, and overall life quality of persons with autism (67). Cross-sectional studies show that parental attitude and coping behavior can also significantly impact the well-being of persons with autism and their families (68, 69). Hayes and Watson (70) meta-analysis identifies parental stress as being significantly greater for parents of autistic children than neurotypical children. Understanding the child–parent relationship is crucial for improving their social skills, emotional health, and overall quality of life (69).

Cluster 2(Green) ASD Prevalence and Etiology with keywords including *neurodevelopment disorders, prevalence, epidemiology, genetics, development delay, pregnancy, pediatrics, risk factors, comorbidity, attention deficit hyperactivity disorder (ADHD), intellectual disability,* and *epilepsy*.

This cluster examines the prevalence, distribution, and epidemiological factors related to ASD in children and students, including risk factors, geographic variations, and population studies. The research in this cluster provides epidemiological data to inform public health policies, resource allocation, and service planning for individuals with ASD. Epidemiological studies directly contribute to understanding the prevalence of a condition. In the USA, where screening and diagnostic services are more accessible than in many countries, research evidence suggests that around 1 in 36 children were diagnosed with ASD (16).

While epidemiological studies of ASD highlight prevalence and risk factors, the pathophysiology is key to understanding the development through biological processes and interactions. The pathophysiology of ASD is not well understood, but it is believed to involve both environmental and genetic factors (71).

Longitudinal studies, pregnancy cohort studies, and sibling cohort studies are also important in understanding the development and progression of autism, particularly during the prenatal and infant stages (72). Metabolic conditions (MC) during pregnancy may be broadly linked with an increased likelihood of developmental setbacks and ASD (73). There exists a high risk of ASD associated with familial members implying a causal link with genetics, and with rapid advances in technology associated with genetic identification, there is an increase in research studies investigating common autism genes (74, 75). Mental and physical co-occurring conditions are frequently reported with ASD, with common examples including epilepsy, ADHD (76–78), and intellectual disability (63). This cluster has implications for healthcare providers, policymakers, and researchers.

Furthermore, this cluster encompasses a significant focus on the exploration of biological markers in ASD. Key aspects such as genetics, neurodevelopmental disorders, developmental delay, and comorbid conditions like epilepsy and ADHD are integral to this pursuit. The inclusion of genetics as a keyword denotes an interest in identifying genetic markers that may predispose individuals to ASD. Additionally, the investigation of developmental delays and neurodevelopmental disorders within this cluster highlights the search for biological indicators that may predict or signify ASD. This research is vital for elucidating the complex interplay between genetic, neural, metabolic, and molecular systems in ASD, potentially leading to more precise diagnostic tools.

Cluster 3(Blue) ASD and Early Identification and Intervention with keywords *classification, diagnosis, screening, early detection, early identification, early diagnosis, early intervention, screening, DSM*-5, *machine learning, infants, toddlers* and *validity*.

This research cluster explores methods and tools in ASD diagnosis and screening, assessing their accuracy and emphasizing early detection and intervention for improved ASD outcomes. It examines early behavioral signs, screening instruments, and intervention methods that could shape developmental paths and tackle ASD-related issues.

The diagnostic process uses algorithms and models to interpret ASD-related data, improving the screening and diagnosis procedures (79), and impacting healthcare workers, educators, and policymakers in standardizing ASD diagnostic tools. Early autism diagnosis in children is key for successful intervention and improved outcomes (64, 67). Timely detection and appropriate support can bolster adaptive behaviors in autistic children and aid social adaptation.

Diagnosis of ASD is carried out through a neuropsychological evaluation, which assesses language, attention, motor performance, and social skills (80, 81). Screening tools like the Childhood Autism Rating Scale (CARS-2) (82) and psychiatric status rating scales are widely used for autism assessment in preschool and toddler-aged children, offering high sensitivity and specificity (83, 84). Technological advancements are expanding our understanding of autism and its underlying mechanisms, thereby improving support and life quality for ASD individuals. Machine learning and classification algorithms have improved diagnostic imaging and early autism diagnosis (62, 85). MRI has detected brain differences in autistic infants (86), and eye-tracking technology is used to study eye movements and gaze patterns, identifying atypical visual attention and social interaction patterns associated with ASD (87). More recently, the culmination of a seminal research line started in 2008, on eye-tracking and clinical diagnosis of autism, was announced early in 2023 (88, 89). Furthermore, virtual reality (90) has shown promise as an innovative therapeutic tool in treating autism.

Cluster 4(Yellow) ASD and Mental Health with keywords: *adolescents, anxiety, child, depression, mental health,* and *sleep*.

This cluster examines the relationship between mental health conditions and ASD, including emotional well-being, and associated conditions like depression and anxiety, and investigates effective interventions and therapies to support their mental health needs. It explores comorbidity, risk factors, and prevalence. Adolescence can be a tough period for the well-being of young people with autism (91), presenting complex issues that require a multidisciplinary approach. Younger children can also face challenges, including mental health issues; anxiety, depression, and sleep disorders appear most significantly in research to date (92). On average, research evidence shows a prevalence of anxiety in 30–40% of autistic children and adolescents (93, 94), which includes a range of specific anxiety conditions; 11% for depressive disorders and 13% for sleep disorders (93). This has implications for healthcare providers, educators, and mental health practitioners in developing integrated approaches for addressing mental health needs in individuals with ASD.

### ASD research and SDG

RQ7 of the study was to understand ASD research in the context of SDG. Studies in the past also have tried to understand an area of research in the context of SDG (95). Although ASD is not explicitly referred to in any SDG, it is implicit in the term “inclusion” in several SDGs. Table 6 displays the distribution of total publications (TP) and total citations (TC) across the various SDGs.

**Table 6:** ASD research mapped to SDG.

SDG	TP	TC	% share
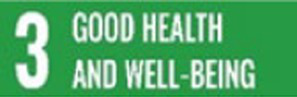	6,124	195,057	50.8%
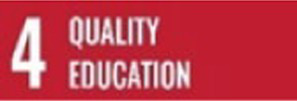	94	961	0.78%
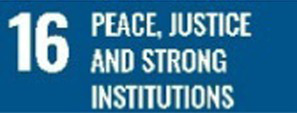	75	1,091	0.62%
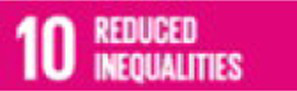	33	453	0.27%

TP, total publications; TC, total citations.

We further review the highly cited ASD publications mapped to SDG (Table 7).

**Table 7:** Highly cited ASD publications mapped to SDG.

Title	Journal	Year	References	TC	SDG mapping
Prevalence of autism spectrum disorder among children aged 8 years—Autism and developmental disabilities monitoring network, 11 Sites, United States, 2014	MMWR Surveillance Summaries	2018	(96)	2,596	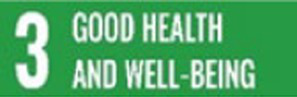
Identification of risk loci with shared effects on five major psychiatric disorders: A genome-wide analysis	The Lancet	2013	(97)	2,175	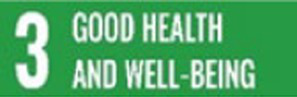
Prevalence of autism spectrum disorder among children aged 8 years-autism and developmental disabilities monitoring network, 11 Sites, United States, 2016	MMWR Surveillance Summaries	2020	(98)	1773	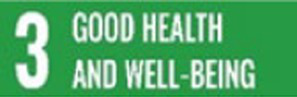
The contribution of *de novo* coding mutations to autism spectrum disorder	Nature	2014	(99)	1,665	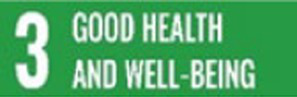
Sporadic autism exomes reveal a highly interconnected protein network of *de novo* mutations	Nature	2012	(100)	1,640	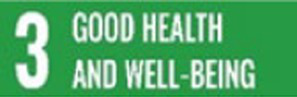

TC, total citations.

The Autism and Developmental Disabilities Monitoring (ADDM) Network offers ASD prevalence data for eight-year-old children across various U.S. communities (96). This report updates ASD prevalence estimates and characterizes the population of children with ASD. The prevalence is reported to be higher than past estimates and varies across racial/ethnic groups and communities, signifying a demand for behavioral, educational, and occupational services.

Zablotsky et al. (101) study used the National Health Interview Survey to assess the prevalence of 10 developmental disabilities among children in the United States between the ages of 3 and 17. The prevalence of any developmental impairment, ADHD, autism spectrum disorder, and intellectual disability has been found to have significantly increased between 2009 and 2017, in contrast to a drop in other developmental delays. Boys, older children, non-Hispanic white and Hispanic children, children with private insurance, children with higher birth weights, children living in cities, and mothers with less education all showed increased incidence. These results tie to SDG 3 (Good Health and Wellbeing) and SDG 4 (Quality Education), underlining the importance of addressing children’s health needs and providing equal educational opportunities.

Autism is frequently diagnosed late, missing key treatment periods. Daniels and Mandell (102) analyses factors in autism diagnosis age and proposes methods to enhance early detection. Findings indicate diagnosis age ranges from 38 to 120 months. Early diagnosis is linked to higher symptom severity, socioeconomic status, parental concern, and family interaction with health and education systems, local resources and state regulations. The study, aligned with SDG4, recommends enhancing parent and provider education, streamlining diagnosis processes, and focusing on underserved groups.

Solmi et al. (103) studied the timing of onset for mental disorders, advocating for mental health promotion and prevention, aligning with SDG 3 (Health and Wellbeing). The research underscores early intervention’s significance and the need to revise the mental health system, particularly the child/adult service divide at age 18, advancing SDG 3’s universal mental health care access.

The study by Lavelle et al. (104) explored the relationship between ASD diagnoses and their impact on service use, caregiver time, and cost outcomes. Results showed that children with ASD had higher healthcare and educational services costs. However, the parents of children with ASD did not incur higher out-of-pocket costs or spend more time on caregiving activities. The study highlights the substantial economic burden of ASD, which extends beyond healthcare. It underlines the importance of addressing the needs of those affected by the disorder, aligning with SDG 3.

### ASD research and COVID-19

It is interesting to understand the impact of COVID-19 on research related to ASD to answer RQ8 (105). The loss of essential medical and educational services during the COVID-19 pandemic, as well as the need for assistance for people with intellectual and developmental impairments, including those with ASD, were both identified by a large-scale global study (106). Loneliness, a significant factor affecting mental health in those with ASD (107), was exacerbated during the lockdown. The quarantine period has caused stress in some adults (108), and many have been affected by severe difficulties, such as losing a job or a loved one, which has had a negative impact on their quality of life. The mental distress of parents and caregivers can increase the child’s mental health risk, as the parent’s well-being can affect the child’s mental health (109). The use of telehealth and technology-based approaches, including video observations, interactive live video evaluations, and phone or online methods, showed promise in assessing children during the pandemic and could potentially continue to provide remote families access even beyond the pandemic (34). Parent’s well-being can affect the child’s mental health (109). The use of telehealth and technology-based approaches, including video observations, interactive live video evaluations, and phone or online methods, showed promise in assessing children during the pandemic and could potentially continue to provide remote families access even beyond the pandemic (34, 110).

**Figure 8:**
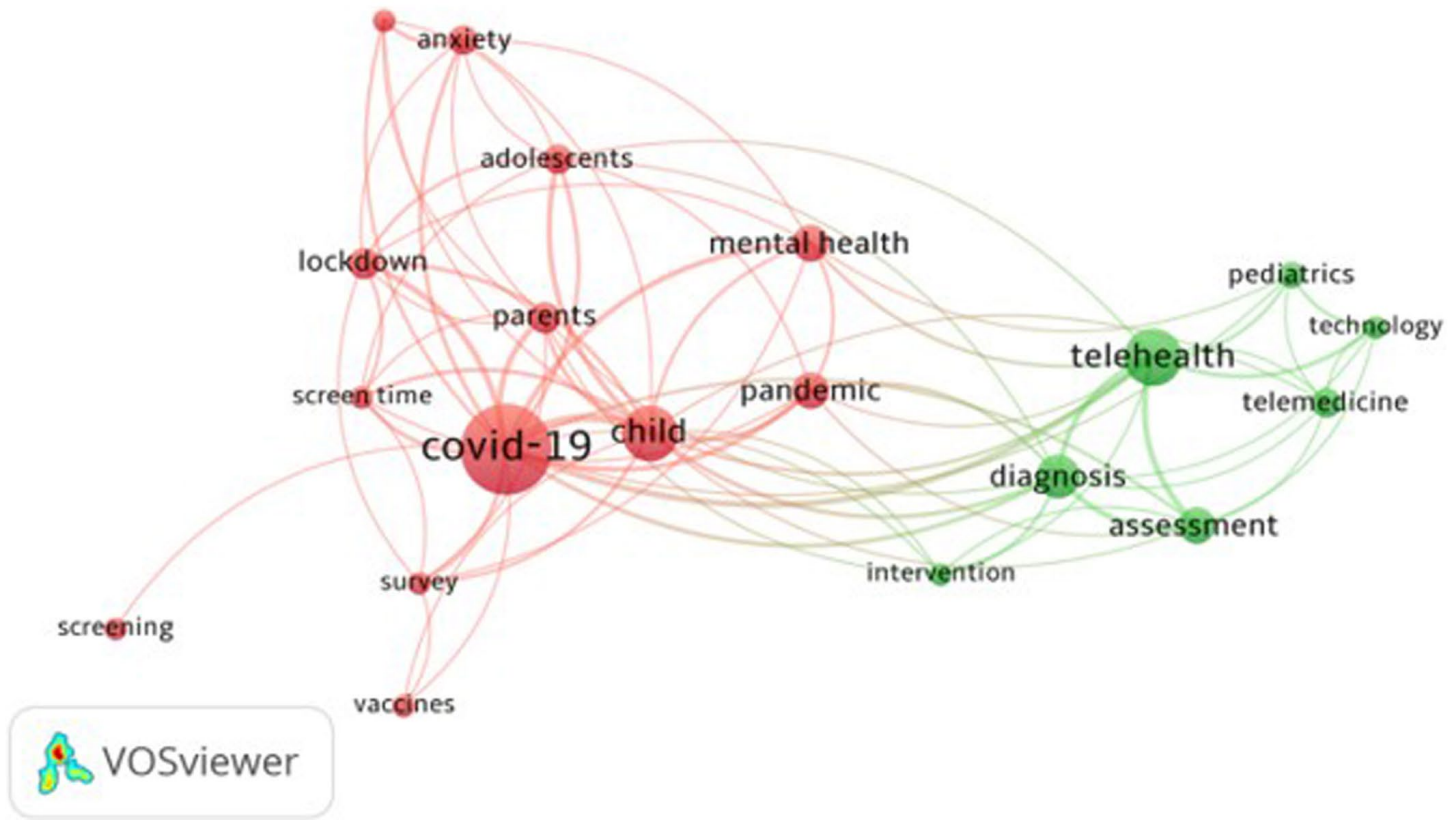
Keyword co-occurrence network of ASD and COVID-19.

The keyword co-occurrence network of ASD and COVID-19 shows two main clusters (Figure 8), representing keywords by their label and circle. The first cluster (Red) has the theme of ASD and Mental Health. The focus is on the impact of COVID-19 on the well-being and mental health of autistic children, adolescents, and their families, with the lockdown experience. The second cluster (Green) on the theme of ASD and Telehealth highlights the change to online service delivery that cascaded out of COVID-19 regarding access to screening and diagnosis and other support services previously delivered face-to-face.

The most cited five studies which explore ASD and COVID-19 include Jeste et al. (106), Türkoğlu et al. (111), Ellison et al. (112), Lugo-Marín et al. (113), and Cahapay (114).

Jeste et al. (106) USA and international study, published by the Journal of Intellectual Disability Research and set during the COVID-19 restrictions, explored the changes that were made to educational and healthcare services for families of people with intellectual and developmental disabilities. Service provision was greatly affected for most of the 808 families, although when available telehealth proved effective at meeting some needs. Türkoğlu et al. (111), studied the relationship between sleep patterns and symptom severity for autistic children during lockdown, proposing some beneficial effects of managing sleep patterns. Ellison et al. (112) systemic review of telehealth services for autistic children and adolescents suggested that the benefits of remote services can outweigh face-to-face services, with more conclusive results still emerging. Lugo-Marín et al. (113) investigated the impact of lockdown and social distancing, including mental health of autistic individuals and their caregivers. Cahapay (114) carried out a qualitative study with 5 case studies of parents who educated their autistic children at home during COVID-19.

## Conclusion

Overall, there has been a steady growth in ASD research in screening over the past 12 years. Open Access journals have also increased steadily from 2013 attracting higher citations per paper, suggesting higher quality and reputation. There is a good representation of first-authored female publications, increasing from about 56.6% in 2011 to 65% in 2022. The top countries contributing to ASD research are the United States and the United Kingdom. Harvard University is the leading contributing institution, and the University of North Carolina has the highest TC/TP ratio. The Journal of Autism and Development Disorders is the top journal in this area, while MMWR Surveillance Summaries received the most online attention.

The significant increase in the prevalence of diagnosed ASD among school-aged children in the US, as reported by the National Survey of Children’s Health, raises important public health concerns (115). The greatest increase was observed among boys and adolescents aged 14–17, suggesting that more previously unrecognized cases are being diagnosed. Some of the factors that could account for the increase in numbers include better recognition and identification of autism (15); greater awareness of parents and other professionals; better access to diagnosis; and refinement of the diagnostic process (60, 116).

The keyword co-occurrence analysis outlined four fundamental research themes within ASD. The first theme, Child Development and Support Systems highlights the specific areas of child development impacted by ASD and emphasizes the need for targeted support systems. The second theme, Prevalence and Etiology, explores the rates and root causes of ASD, incorporating risk factors, geographic differences, and insights from population studies. The third theme, Early Identification and Interventions, underscores the paramount importance of timely, accessible and affordable (117) ASD diagnosis and interventions for enhancing autistic children’s quality of life and overall well-being. Finally, the Mental Health theme delves into the psychological challenges faced by children, and particularly adolescents, living with autism, such as anxiety, depression, and sleep disorders. These themes underscore the multifaceted nature of ASD and the imperative for a broad and deep-seated approach to its research.

Worldwide, the detection of autism is on the rise (15, 118). Barriers to diagnosis include community knowledge, perceptions of autism, parental knowledge and awareness, and issues within the healthcare system (119). Analyzing prevalence data is essential in understanding the impact of various factors such as research intensity, healthcare accessibility, and socio-cultural influences on the detection and reporting of conditions. Although there appears to have been a marked increase over time in prevalence data in the West, in particular the USA (15), Europe (120), and the UK (19), the actual figures vary significantly across countries and different socioeconomic groups.

Delays in diagnosis of ASD are a significant public health issue because they frequently lead to missed opportunities for early intervention during a crucial developmental stage. According to Daniels and Mandell (121) analysis of 42 peer-reviewed publications, the average age of ASD diagnosis is between 38 and 120 months old and has been dropping over time. Greater symptom intensity, high socioeconomic position, and parental concern are all linked to early diagnosis. Early ASD screening involves family contact with the health, school, and community resources systems. It is essential to improve parental and provider education on early developmental problem awareness, shorten the procedure from initial concern to diagnosis, and focus on underprivileged communities in order to solve this public health issue. Early detection efforts will help ensure that children with ASD receive the necessary support and services for optimal development and well-being (122).

A systematic review of the impact of COVID-19 on autism suggests a need to develop evidence-based screening and assessment tools for remote use (34) and rural communities (123). The recent impact of COVID-19 on social research (124), and our analysis shows that the key areas of attention during COVID-19 for persons with autism were mental health, telehealth, and access to education and health services.

Contributions relating to SDG of ASD publications were primarily under “Good Health and Wellbeing” (SDG 3), highlighting the importance of addressing the health needs of individuals with ASD and promoting their well-being. Early intervention and comprehensive mental health services are crucial for better outcomes. The effect of the SDG is likely to significantly increase ASD research from an international perspective in the long term.

## Recommendations

These recommendations adopt a dual strategy offering research and service delivery guidance. While biomedical research for early identification remains a priority, there’s an opportunity to balance the allocation of funding for autism research by including a focus on service delivery and awareness raising.

- Prioritize early identification and intervention: Our analysis indicates that the early screening and diagnosis increase is largely concentrated in certain countries and economic groups. More comprehensive and quality research leads to better identification and reporting of cases. Encourage interdisciplinary research, integrating insights from psychologists, educators, medical professionals, and researchers. The prioritization of early identification and intervention is crucial, as our study demonstrates that current efforts in early screening and diagnosis are predominantly concentrated in specific countries and economic groups. This finding underscores the need for broader and more inclusive early intervention strategies globally. Considering the pivotal role of technological advancements in current ASD research, advocating for technologies such as ASD telehealth screening and therapy is essential, especially in underserved communities, to make diagnosis and treatment more accessible.
- Expanding ASD Interdisciplinary Research: This recommendation for interdisciplinary research is based on our findings highlighting the increasing diversity in ASD research authorship. This diversity necessitates a collaborative approach that integrates insights from multiple disciplines, including psychology, education, and medical research. However, the analysis reveals a disparity in ASD research focus, often overlooking lower socioeconomic regions. Expanding research and also including include socioeconomic regions is vital for informing policy at National levels and ensuring a holistic approach to ASD.
- Increase research on biological markers: Research in screening informs and improves diagnostic criteria and healthcare practices, which in turn affect prevalence rates. The emphasis on researching biological markers stems from our findings (Cluster ASD Prevalence and Etiology) and on the early onset of neurodevelopmental disorders. Identifying early-stage biological indicators, such as genetic or neural markers, is key for developing affordable diagnostic systems and facilitating early intervention. The early onset of neurodevelopmental disorders suggests that biological indicators could present at an early stage, such as genetic, neural, metabolic, and molecular systems (14, 125). Research to create affordable systems based on these biomarkers or external observations could lead to clearer diagnoses and promote early intervention. Identifying early-stage biological indicators, such as genetic or neural markers, is key for developing affordable diagnostic systems and facilitating early intervention. Research can uncover the underlying causes of a condition, including environmental, genetic, and lifestyle factors, which are crucial in understanding variations in prevalence across different regions.
- Prioritize Mental Health and Support Services: Emphasize research to support the development of mental health services tailored to meet the needs of individuals with ASD, including addressing co-occurring conditions which include anxiety, depression, and sleep disorders. The prevalence of co-occurring mental health conditions with ASD, as identified in our research, highlights the need for tailored mental health services and enhancing access to various support services for individuals with ASD and their families. Develop and research the efficacy of online training for healthcare providers, educators, and parents to recognize early signs of ASD.
- Improve Disability Inclusion: Our findings indicate a gap in Disability Inclusion within the field of ASD. Emphasizing this area could lead to the development of more inclusive disability-specific programs and support services for individuals with ASD and their families, including therapy, counseling, and educational resources. The need for enhanced societal understanding and acceptance of ASD, underscores the importance of investing in public awareness campaigns to reduce stigma and facilitate social integration.

## Limitations

While this study offers valuable insights into ASD research through bibliometric methodology, it has inherent limitations. Firstly, bibliometric reviews can only show the short-term impact of research (126). Secondly, the study relied on the Scopus database, which, although comprehensive, may contain errors and is constantly being updated with new publication data, affecting the study’s results. The choice of search terms and keywords used in the database can also be limiting, as it may not capture all relevant information on the topic of study. Additionally, network visualization, used in this study to analyze countries, journals, and keywords related to ASD, can simplify the data but may result in loss of information. An example of this is “gender” which did not come up in the thematic analysis, however, a manual sifting of abstracts revealed 661 articles referring to gender, 77 of these were specifically focused on the disparities between the number of males and females diagnosed and the gender ratio for ASD. With consistent reporting of a male: female gender ratio of 3:1 (23, 59) proposed justifications for this include bias in the diagnosis process and diagnostic tools, as well as a tendency for autistic girls and women to mask and conceal their autistic traits (127).

However, despite these limitations, this study still adds valuable insights to the existing body of research on ASD.

## Data availability statement

The original contributions presented in the study are included in the article/supplementary material, further inquiries can be directed to the corresponding author.

## Author contributions

PN: Conceptualization, Formal analysis, Methodology, Writing – original draft, Writing – review & editing. SS: Writing – original draft, Writing – review & editing. MS: Writing – review & editing. VK: Visualization, Writing – original draft. AM: Writing – original draft. RR: Conceptualization, Methodology, Project administration, Supervision, Visualization, Writing – original draft, Writing – review & editing.
